# Substrate-Independent, Regenerable Anti-Biofouling Coating for Polymeric Membranes

**DOI:** 10.3390/membranes11030205

**Published:** 2021-03-13

**Authors:** Juan Zhang, Guang Wang, Jianhua Zhang, Zhiguang Xu, Yan Zhao, Yichao Wang, Fenghua She, Stephen Gray, Lingxue Kong

**Affiliations:** 1 Institute for Frontier Materials, Deakin University, Geelong, VIC 3216, Australia; victorwang00@hotmail.com (G.W.); zhiguang.xu@mail.zjxu.edu.cn (Z.X.); Yichao.w@deakin.edu.au (Y.W.); mary.she@deakin.edu.au (F.S.); 2 Institute for Sustainable Industries and Liveable Cities, Victoria University, Melbourne, VIC 8001, Australia; jianhua.zhang@vu.edu.au (J.Z.); stephen.gray@vu.edu.au (S.G.); 3 College of Textile and Clothing Engineering, Soochow University, Suzhou 215123, China; yanzhao@suda.edu.cn

**Keywords:** polymeric membrane, anti-biofouling, silver nanoparticles, re-generable

## Abstract

Biofouling is a common but significant issue in the membrane process as it reduces permeate flux, increases energy costs, and shortens the life span of membranes. As an effective antibacterial agent, a small amount of silver nanoparticles (AgNPs) immobilized on membrane surfaces will alleviate the membrane from biofouling. However, loading AgNPs on the membrane surface remains a challenge due to the low loading efficiency or the lack of bonding stability between AgNPs and the membrane surface. In this study, a substrate-independent method is reported to immobilize silver nanoparticles on polymeric membrane surfaces by firstly modifying the membrane surface with functional groups and then forming silver nanoparticles in situ. The obtained membranes had good anti-biofouling properties as demonstrated from disk diffusion and anti-biofouling tests. The silver nanoparticles were stably immobilized on the membrane surfaces and easily regenerated. This method is applicable to various polymeric micro-, ultra-, nano-filtration and reverse osmosis (RO) membranes.

## 1. Introduction

Membrane-based separation technology has become a well-known commercial method over the past decades for water treatment and desalination. However, membrane fouling, especially biofouling, remains one of the major obstacles affecting efficiency, maintenance, and the lifespan of membranes in water and wastewater treatment [1,2,3,4].

Membrane biofouling refers to the undesirable accumulation of microorganisms on the membrane surface that reduces the permeate flux, increases energy costs, and shortens the lifespan of the membranes [5,6,7,8]. Membrane biofouling can be very difficult to control due to the self-replicating nature of microbes and is often considered irreversible [9,10]. Therefore, there is a critical need to develop biofouling control strategies that can lower the biofouling potential inside membrane modules during filtration by continuously inactivating bacteria and suppressing biofilm formation [11].

Silver is known as an effective antibacterial agent due to its excellent antibacterial properties against numerous types of bacteria and low toxicity to mammals [12,13,14,15]. However, silver usage as biocide in membrane processes is limited, mainly because of its relatively high cost and extremely low efficiency. This is because only a small fraction of the silver nanoparticles (AgNPs) from the directly AgNP embedding membrane fabrication process are exposed to microbes [16,17,18,19,20]. Furthermore, AgNPs can only be embedded inside the membrane during membrane fabrication, and thus there is no opportunity for AgNPs to be recharged after leaching.

As the top layer (active layer) of membranes plays a vital role in separation performance and is where biofilm develops, immobilizing a small amount of silver on the membrane surface will protect the membrane from biofouling. However, loading AgNPs only on the membrane surface remains a challenge because of the low loading efficiency or the weak bonding stability between AgNPs and the membrane surface. The previously prepared AgNPs could covalently be bonded to chemically modified membrane surfaces [21,22,23]. However, this method involves AgNP synthesis and the use of a capping chemical agent, which adds to the cost. Furthermore, only a fraction of the synthesized nanoparticles in the solution eventually binds to the membrane surface.

The absorption of ionic silver on the membrane surface followed by the in-situ formation of AgNPs has the potential to increase bonding efficiency and improve bonding stability. Zhu et al. chelated ionic silver or metallic silver onto a chitosan-based membrane through the amino groups in chitosan [24]. Cao et al. introduced AgNPs on the surface of a sulfonated polyethersulfone membrane by using vitamin C as a reducing agent [25]. Yang et al. and Ben-Sasson et al. employed an in situ method to simultaneously fabricate and load AgNPs on the polyamide reverse osmosis (RO) membrane surface by chemical reduction [26,27]. However, all these methods are limited to specific membrane materials with functional groups on their surfaces and cannot be applied to other membrane materials. Other studies reported the immobilization of AuNPs with the aid of polyacrylic acid, which was grafted on the membrane surface using gamma-rays [28], the physisorbed free radical grafting technique [29], or the Fenton type reaction [30]. However, the requirements of an inert atmosphere or the introduction of other additional copolymers limits these applications. Developing a facile and more processable method that can immobilize AuNPs on various membrane surfaces will enable membranes to obtain anti-biofouling properties. 

**Scheme 1:**
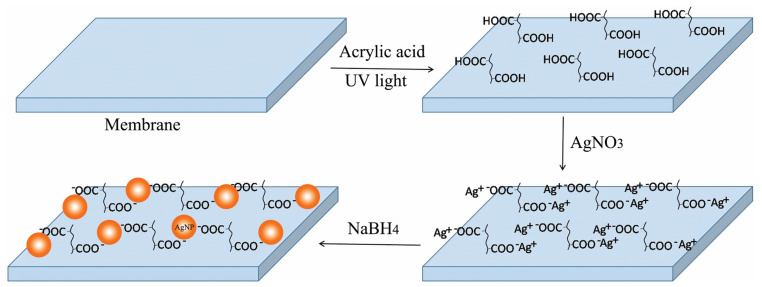
Diagram of in situ formation of silver nanoparticles (AgNPs) on polymeric membranes.

Herein, a substrate-independent method is reported to immobilize silver nanoparticles on polymeric membrane surfaces with the stabilization of carboxylic groups of polyacrylic acid (PAA), which is grafted on the membrane surface through a facile ultraviolet (UV) irradiation process. In detail, as shown in Scheme 1, the membrane surface was functionalized with carboxylic groups by grafting polyacrylic acid (PAA) through UV-induced polymerization. Silver ions were then absorbed onto the membrane surface through ion exchange to form -COO^−^Ag^+^ groups, followed by in situ formation of AgNPs through chemical reduction. Since a thin layer of polymer with functional groups for AgNPs formation can be grafted onto any polymeric membrane surface through UV irradiation using the Norrish II photo initiator, this method is applicable to any polymeric micro-, ultra-, nano-filtration, or RO membrane, such as polysulfone, polyether sulfone, polyvinylidene fluoride, nylon, polystyrene, polypropylene, and polyamide. Furthermore, AgNPs on membrane surfaces can be easily regenerated in situ after being leached out. Membranes incorporated with AgNPs have better hydrophilicity than original polysulfone membranes. Therefore, membranes with AgNPs immobilized on their surface can been improved from two different perspectives: antibacterial from the incorporation of AgNP, and anti-fouling from the higher hydrophilicity. To the best of our knowledge, this method is being reported for the first time. 

## 2. Materials and Methods

### 2.1. Materials

Polysulfone ultrafiltration membranes (PSf, UF30K) were purchased from Pureach Tech Co. Ltd. (Beijing, China). Nylon microfiltration membranes (pore size 0.22 μm) were ordered from Krackeler Scientific (Albany, NY, USA), and polypropylene (PP) microfiltration membranes (pore size 0.3 μm) were sourced from Shanghai Bandao Shiye Co. Ltd. (Shanghai, China). BW30 TFC RO membranes were purchased from Dow Filmtec Corp., Midland, MI, USA. Methyl methacrylate (MMA, 99%), sodium borohydride (NaBH4, 96%), benzophenone (BP, 99%) and silver nitrate (AgNO3, 99%) were purchased from Sigma Aldrich, St. Louis, MO, USA. De-ionized (DI) water was used to prepare all solutions needed in the study. 

### 2.2. Surface Functionalization 

The surface of the polysulfone (PSf) ultrafiltration membrane was grafted with polyacrylic acid (PAA) using UV-induced polymerization. First, 2.5% acrylic acid (AA) was dissolved in benzene phenone saturated DI water. Then the PSf membrane was immersed in an acrylic acid solution and treated under UV light (Noblelight GmbH, UV-A: 350–400 nm, 80 mW/cm^2^ at 365 nm) for 30 min. Finally, the membrane was immersed in a large quantity of DI water to remove unreacted monomers and ungrafted PAA.

### 2.3. In Situ Immobilization of Silver Nanoparticles

The PAA-modified PSf membrane was placed between a Teflon plate and a rubber frame designed to hold the solutions on the active layer side of the membrane. First, an AgNO_3_ solution (1 to 5 mM) was poured onto the isolated active layer for 10 min to facilitate the adsorption of silver ions to the surface. Subsequently, the AgNO_3_ solution was discarded, leaving only a thin layer of AgNO_3_ solution on the membrane surface. Then, a 5 mM NaBH_4_ solution was poured onto the active layer to reduce Ag (+1) to Ag (0) nanoparticles. Sample details are shown in Table 1. Membranes M1, M2, and M3 stand for the membranes prepared using 1, 3, and 5 mM AgNO_3_ respectively, and all the other parameters were kept the same.

AgNPs were also immobilized onto the membrane surfaces of nylon and polypropylene microfiltration membranes and reverse osmosis polyamide membranes using the same PAA grafting and AgNP generation procedures with 5 mM AgNO_3_ and 5 mM NaBH_4_.

### 2.4. Membrane Characterizations

The membrane surface composition was analyzed using attenuated total reflectance infrared spectroscopy (ATR-FTIR) (Bruker Vetex-70 FTIR spectrometer, Germany). Scans for spectrums were recorded in the standard wavenumber range of 600–4000 cm^−1^, at a resolution of 4 cm^−1^, and an average of 32 scans was reported. XPS measurements were performed on a VG-310F instrument, using Al non-monochromatic X-rays (20 kV, 15 mA) with the hemispherical energy analyzer set at a pass energy of 20 eV for peak scans. The contact angle was measured using a contact-angle measurement system (CAM101, KSV instruments Ltd., Helsinki, Finland). 

The membrane surface morphology was characterized using field emission scanning electron microscopy (FESEM) (Supra SEM 55VP, ZEISS, Germany). All the samples were sputter-coated with gold prior to the FESEM imaging observation. For the transmission electron microscopy (TEM) cross-section image, the in situ AgNP modified membrane sample was fixed in an epoxy resin and cut into 80 nm slices using a Leica UC6 microtome. Then, the samples were loaded onto carbon-coated copper microgrids. TEM images were captured at 200 kV on a JEOL JEM-2100 (JEOL, Peabody, MA, USA).

Membrane filtration performances were evaluated using a dead-end filtration system (Sterlitech HP4750, Kent, WA, USA). The membrane effective area was 14.6 cm^2^. For polysulfone ultrafiltration membrane, the water flux and rejection of the membrane was recorded at 0.1 MPa by filtering a 200 ppm bovine serum albumin (BSA) feed solution, and the concentration of the first 30 mL permeate solution was measured using a UV–vis spectrophotometer (Cary 3, Agilent, Santa Clara, CA, USA) at 280 nm. For nylon and PP microfiltration membranes, the water flux was recorded at 0.2 bar by filtering deionized water. For RO membranes, the water flux was recorded at 15 bar by filtering deionized water. 

To determine the total loading of AgNPs on membrane surfaces, membranes (1.5 cm^2^) were dissolved in 2.5 mL 26% concentrated nitric acid (HNO_3_) and then diluted 40 times with deionized water and filtered with a 0.45 μm filter. The silver contents were then analyzed by inductively coupled plasma optical emission spectrometry (ICP-OES, SHIMAZU 9000, Kyoto, Japan). To study the static release of silver from the membranes, composite membranes were cut into rectangular shapes with areas of 12 cm^2^ and were subsequently immersed in 100 mL of the deionized water at room temperature. After one week, the membranes were immersed in 100 mL fresh deionized water. The concentration of silver in the water was analyzed by ICP-OES. To evaluate the depletion behavior of silver in the filtration process, ultrapure water was filtered through the membrane in a dead-end filtration cell (Sterlitech HP4750) at 0.1 MPa for 12 h. The permeate water was concentrated prior to the ICP-OES analysis. 

The antibacterial activity of the prepared membranes was evaluated using the disk diffusion method against Gram-negative bacterium, *Escherichia coli* (*E. coli*), and Gram-positive bacterium, *Staphylococcus aureus* (*S. aureus*). A circular disk of each membrane was then placed on a bacterial agar surface for incubation for 24 h at 37 °C. The inhibition zone formed after 24 h served as an indicator for the antibacterial activity and was recorded using a light microscope (Olympus BX51, Tokyo, Japan). 

Anti-biofouling tests were performed to evaluate the activity of membranes in preventing bacterial adhesion and reproduction on the membrane surface. Small pieces of pristine PSf membranes and membranes with different AgNP contents were immersed into suspensions of *E. coli* or *S. aureus*, respectively. The membrane samples were taken out of the bacterial suspensions after 24 h of immersion at 37 °C. After that, the samples were immersed in 2.5% (*v*/*v*) glutaraldehyde PBS solution to fix the bacteria remaining on the membrane surfaces. Then, the membrane samples were dried in an oven at 80 °C. The specimens were sputter-coated with gold and examined with the FESEM (Supra SEM 55VP, ZEISS, Jena, Germany).

## 3. Results and Discussion

### 3.1. Membrane Surface Composition and Morphology

**Figure 1:**
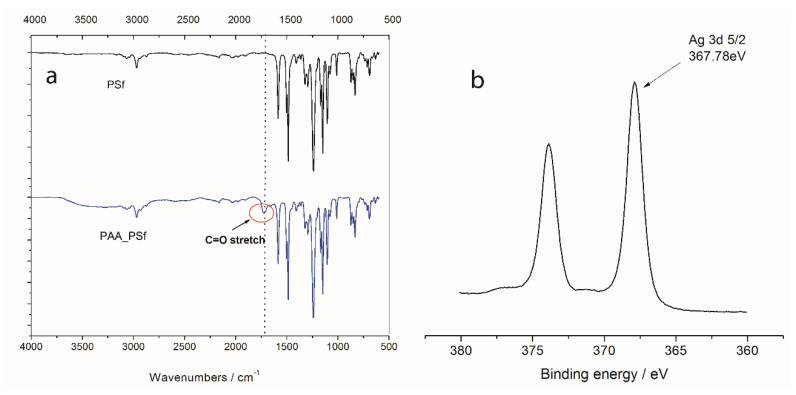
(**a**) FTIR spectra of polysulfone membranes (PSf) and acrylic acid modified polysulfone membranes (PAA_PSf), and (**b**) XPS spectrum for Ag (3d) of sample M2.

Ultra-filtration polysulfone (PSf) membrane was used as a model membrane material. The membrane surface was functionalized with carboxylic groups by grafting polyacrylic acid (PAA) using UV-induced polymerization. Figure 1a shows the FTIR spectra of the pristine PSf membrane and the PAA-modified membrane (PAA_PSf). The appearance of a peak at 1730 cm^−1^ associated with the symmetric vibration of C=O for the PAA-modified membrane (PAA_PSf) indicates that the PAA was successfully grafted onto PSf membrane surface. XPS analysis (Table 2) revealed that the percentage of oxygen on the membrane surface increased from 8.4% for the pristine PSf membrane to 12.2% for the PAA-modified PSf membrane (PAA_PSf).

Previous studies have reported that poly(sodium acrylate) containing carboxylic groups were used as stabilizers for fabricating metal nanoparticles [31,32,33]. In the synthesis of nanoparticles within a polyelectrolyte multilayer thin film, carboxylic groups were employed as “nanoreactors” to bind metal cations from an aqueous solution, followed by chemical reduction to produce nanoparticles [34,35,36]. Here, PAA with carboxylic groups was grafted onto membrane surfaces to act as “nanoreactors” to immobilize silver nanoparticles to enhance membrane anti-biofouling properties. Firstly, PAA-modified membranes were immersed in a AgNO_3_ solution to facilitate silver ion adsorption on the surface to form -COO^−^Ag^+^ groups. Then, the membranes were submerged in a NaBH_4_ solution to reduce Ag (+1) to Ag (0) nanoparticles. 

To further verify the coordination between the carboxylic groups and the silver nanoparticles, the XPS spectra of the membranes were obtained. Figure 1b is a typical Ag (3d) spectrum of the membrane after silver nanoparticle immobilization. The binding energy of ionic silver (3d 5/2) in silver nitrate is 368.21 eV [24], but that of coordinated silver would shift slightly to a lower value. As shown in Figure 1b, the Ag (3d 5/2) peak at 367.78 eV indicates the presence of coordinated silver on the surface, confirming the immobilization of silver onto the membrane. The surface silver content, as determined by ICP-OES, was 3.45%, 5.50%, and 9.06% for membranes M1, M2, and M3, respectively (Table 2), while the membrane without PAA surface modification only had 0.19% silver on the surface. This further supports the proposition that carboxylic groups play an important role in coordinating the silver nanoparticles. 

**Figure 2:**
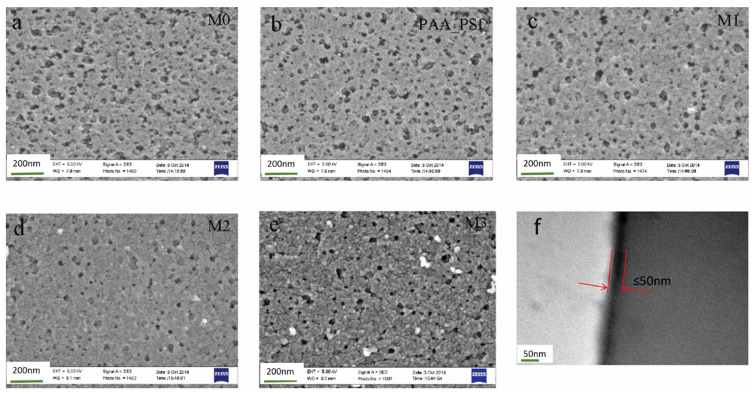
(**a**–**e**) SEM morphologies of the surfaces of membranes; (**f**) TEM micrograph of the cross section of M3 after in situ formation of AgNPs.

Figure 2a–e shows the SEM morphologies of membranes with and without immobilization of silver nanoparticles. The SEM images indicate that after Ag nanoparticle immobilization, membranes M1, M2, and M3 prepared using 1, 3, and 5 mM AgNO_3_, respectively, had reduced pore size and surface porosity compared to the pristine PSf (M0) and PAA-modified membranes (PSf-PAA). TEM results suggests that silver nanoparticles should be less than 50 nm (Figure 2f). The surface thin layer containing PAA and AgNPs with dark color is less than 50 nm, so the AgNP should be less than 50 nm. As membrane cross-sections are about 80 nm thick, the images of AgNPs should overlap with each other, and appear as a continuous film.

### 3.2. Stability of the Immobilized Silver Nanoparticles

**Figure 3:**
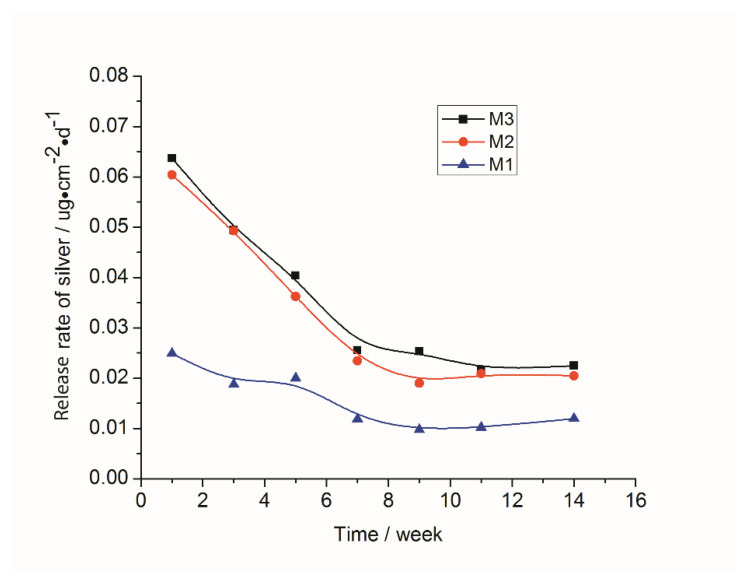
The release rate of silver during the 14 week static leach test.

The stability of the silver nanoparticles immobilized on membrane surfaces was evaluated via both static release and filtration test. ICP-OES was used to analyze the Ag released samples. The total silver loaded on the membrane surfaces was 4.75, 10.88, and 15.20 μg/cm^2^ for M1, M2, and M3, respectively (Table 3). Figure 3 shows the silver release rate during a 14 week static leach test. The initial silver release rates from the M1, M2, and M3 were approximately 0.025, 0.06, and 0.064 μg·cm^−2^·d^−1^, respectively, and then declined gradually with time. After 7 weeks, the silver release rates for M1, M2, and M3 were reduced to around 0.01, 0.02, and 0.024 μg·cm^−2^·d^−1^, respectively, and stabilized. The leach results suggest that membrane antimicrobial effects could persist for a long time, and based on the steady release rates, the antimicrobial properties of these membranes could be expected to be effective for over a year. The silver release rate was much lower compared to membranes incorporated with the previously prepared AgNPs [21], in which a silver release rate of 0.1 μg·cm^−2^·d^−1^ was observed with a membrane total silver loading of 15.57 μg·cm^−2^. 

In filtration tests, silver ion concentrations in the permeate were very low, being less than 1 ppb in the initial 12 h of the test. According to the WHO guideline [37] for drinking water, the Ag threshold is limited to 100 ppb. From this point of view, it is unlikely any health concerns will arise from using silver immobilized membranes for membrane biofouling disinfection, since the Ag released was much lower than the WHO threshold, even during the initial stages of filtration. However, further testing upon scale-up is required to confirm this result. 

### 3.3. Restoration of Silver Nanoparticles

**Figure 4:**
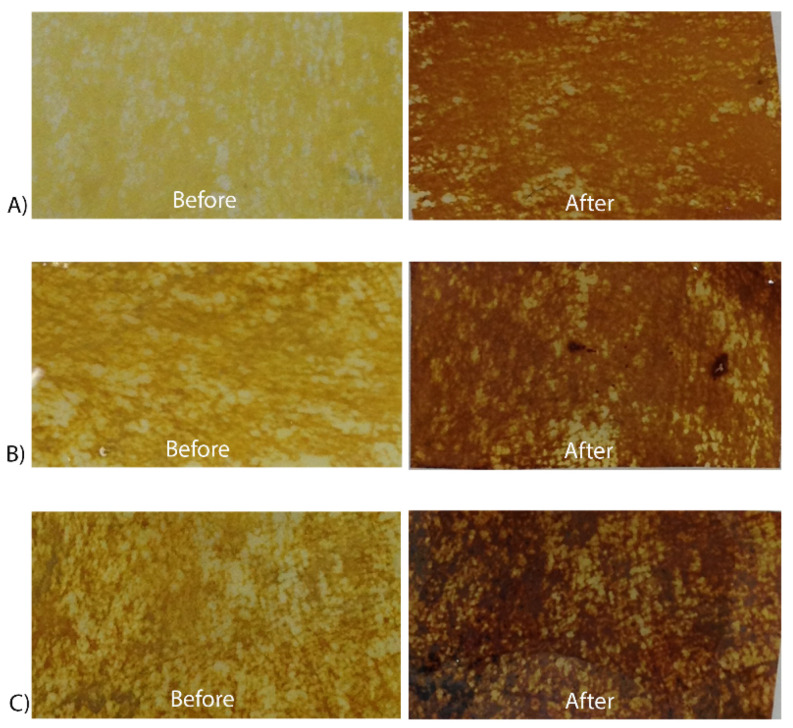
The surface color of membranes (**A**) M1, (**B**) M2 and (**C**) M3 before and after silver nanoparticle regeneration.

After the AgNPs have leached out, the anti-fouling and anti-bacterial properties will be lost, so the restoration of silver nanoparticles is important. AgNPs can be easily restored in situ by simply immersing membranes in AgNO_3_ solution and then NaBH_4_ solution. To demonstrate this, membranes after a 14 week leach test were used to restore AgNPs on their surfaces. The sign of successful restoration of AgNPs on membrane surfaces was a change of the membrane’s surface color from yellow-brown to a darker yellow-brown (Figure 4). Higher concentrations of the AgNO_3_ solution resulted in a darker color, indicating a higher loading of silver on the membrane. The total silver on the membrane surfaces after restoration was 8.12, 13.9, and 19.32 μg/cm^2^ for M1, M2, and M3, respectively (Table 3), confirming that AgNPs were successfully re-immobilized on the membrane surfaces. With the regeneration of AgNPs, the antimicrobial effects were recovered. 

### 3.4. Surface Hydrophilicity

Contact angle measurements were performed to measure the surface hydrophilicity, and the results are shown in Table 4. The pristine PSf membrane (M0) had a contact angle of 62.0°, and the PAA-modified membrane (PAA_PSf) had a contact angle of 55.1°, indicating that the membrane after PAA modification became more hydrophilic. Membranes loaded with different amounts of Ag had slightly smaller contact angles than that of the PAA-modified membrane, suggesting that the AgNP immobilization slightly changed the hydrophilicity of the membrane relative to the PAA modification membrane. The enhanced hydrophilicity of membranes after AgNPs incorporation should result in better anti-fouling properties. Therefore, the membranes with AgNPs immobilized on their surfaces could have improved performances from two different perspectives: AgNP, resulting in improved antibacterial properties; and higher hydrophilicity, resulting in improved anti-fouling properties.

### 3.5. Water Permeability and BSA Rejection

The decline in membrane permeability observed during filtration of BSA and BSA rejection are shown in Table 4. The membrane permeability (A) decreased from 131.1 L·m^−2^·h^−1^·bar^−1^ for the pristine membrane to 63.2 L·m^−2^·h^−1^·bar^−1^ for the PAA-modified membrane (PAA_PSf). The membrane permeability decreased further to 40.0, 36.2, and 36.8 L·m^−2^·h^−1^·bar^−1^ for membranes M1, M2, and M3 immobilized with AgNPs. However, the BSA rejection increased from 75.8% for the pristine membrane to 89.8% for the PAA-modified membrane and increased further to 92.3%, 90.3%, and 91.7% for membranes M1, M2, and M3, respectively. The decrease in membrane permeability and increase in BSA rejection were attributed to the grafted thin layer of PAA and the deposited AgNPs on the membranes, which reduced the membrane surface porosity and pore size as seen from SEM images (Figure 2). Therefore, for UF membranes to achieve the desired permeability following surface modification, it is recommended to use unmodified pristine membranes with bigger pore size and higher porosity than ultimately required. 

In order to investigate the effects of deposition of AgNPs on the permeability of different membranes, the permeability of PP and nylon microfiltration, and polyamide RO membranes immobilized with AgNPs using the method described in Section 3.7, were also studied (Table 5). For microfiltration of PP membranes, the permeability of the membrane immobilized with AgNPs (623 L·m^−2^·h^−1^) was slightly higher than the pristine membrane (616 L·m^−2^·h^−1^). This is in agreement with a previous study showing that PP membranes grafted with thiol groups and immobilized with silver had a higher water flux than the pristine membrane due to the improved hydrophilicity after grafting of the thiol group and silver immobilization [38]. The permeability of the nylon microfiltration membrane immobilized with AgNPs (274 L·m^−2^·h^−1^) was much lower than that of the pristine membrane (484 L·m^−2^·h^−1^). This effect is similar to the polysulfone ultrafiltration membrane. The deposition of AgNPs on the polyamide RO membrane surface changed the membrane permeability from 39.3 L·m^−2^·h^−1^ for the pristine membrane to 33.6 L·m^−2^·h^−1^ for the membrane with AgNPs (about a 15% decrease), which is in agreement with the literature [27]. These results indicate that the immobilization of AgNPs on different membrane surfaces will have different impacts on their membrane permeability. For RO membranes with high resistance, the addition of a thin coating of AgNP particles has little effect on its permeability, but for larger pore size micro-filtration and ultra-filtration membranes of lower resistance, the addition of a AgNP layer has a more significant effect on its permeability. For these lower-resistance membranes, changes in surface wetting properties of the AgNPs may also result in significant permeability modifications.

### 3.6. Anti-Biofouling Performance

**Figure 5:**
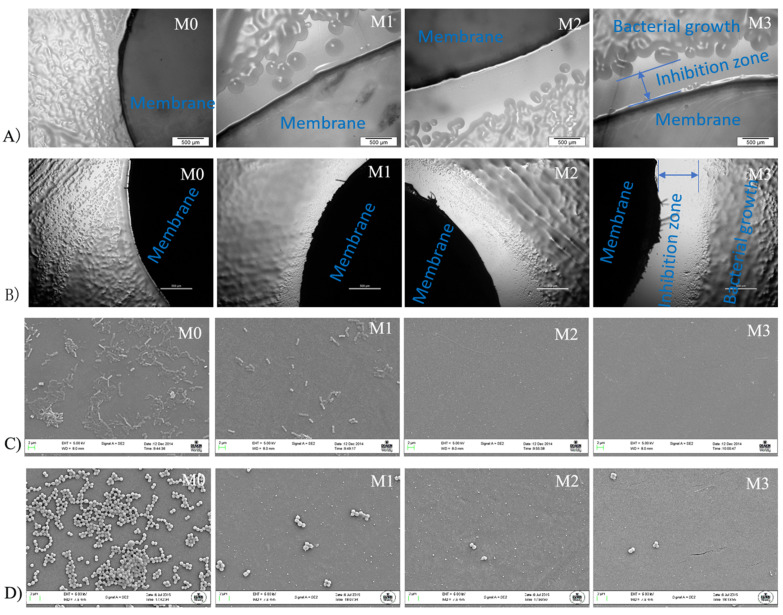
Antibacterial and anti-biofouling results of the membrane surfaces using the disc diffusion method against (**A**) *E. coli* and (**B**) *S. aureus*; and biofilm formation test against (**C**) *E. coli* and (**D**) *S. aureus*.

In order to verify that immobilized silver nanoparticles on membrane surfaces have a broad-spectrum antibiotic effect, both *Escherichia coli* (*E. coli*) as a typical Gram-negative bacterium and *Staphylococcus aureus* (*S. aureus*) as a typical Gram-positive bacterium were used, as the two types of bacteria are commonly found in water and wastewater. The disk diffusion method [13] and bacterial suspension immersion experiment (biofilm formation test) were performed to evaluate the membrane anti-biofouling properties of the AgNP immobilized membranes. The disk diffusion test results shown in Figure 5A,B indicate that the AgNP immobilized membranes (M1–M3) had a significant inhibition capacity against *E. coli* and *S. aureus*. There was no inhibition zone around the pristine PSf membrane (M0), suggesting that it had no inhibition toward the growth of both *E. coli* and *S. aureus*. This indicates that antibacterial activity was caused by the AgNPs and not by the PSf membrane. All AgNP immobilized membranes showed significant inhibition effects, and with an increase in AgNPs content, the inhibition zone became clearer and wider. 

The anti-biofouling tests were investigated with 24 h immersion in bacterial suspension to evaluate the anti-biofouling property of the membranes. The results are shown in Figure 5C,D. Bacterial colonies formed on the surface of the pristine membrane (M0); therefore, the pristine membrane appeared to be prone to biofouling by both *E. coli* and *S. aureus*. The membranes immobilized with AgNPs (M1–M3) appeared to be very effective at preventing the initiation of bio-films, and only a few cells were found on the membrane surfaces. As the AgNP content increased, fewer bacteria attached to the membrane surfaces. If membrane surfaces are able to limit the growth and adhesion of bacteria, it would slow down the formation of a fouling layer. In summary, AgNPs immobilized on membrane surfaces should lead to good anti-biofouling performance.

### 3.7. Immobilization of AgNPs on Different Membrane Surfaces

**Figure 6:**
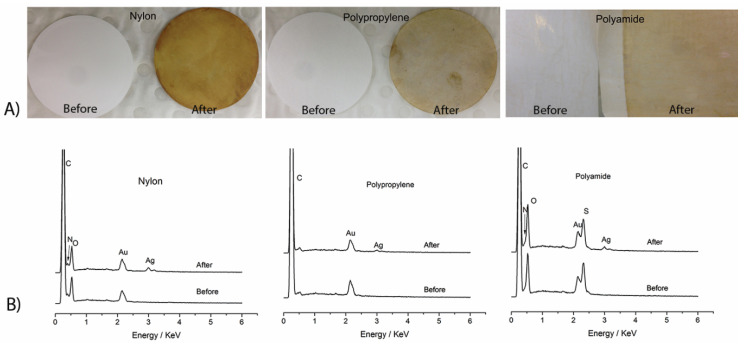
The surface color (**A**) and SEM-EDS (**B**) of nylon, polypropylene (PP), and polyamide membranes before and after silver immobilization.

This technique of immobilizing AgNPs on membrane surfaces has the potential for very wide applications with different membranes. For example, AgNPs were immobilized onto the membrane surfaces of nylon and polypropylene microfiltration membranes and a reverse osmosis polyamide membrane. Figure 6A shows the surface colors of the nylon, PP, and polyamide membranes before and after silver immobilization. The change in membrane surface color for all these membranes from white for the pristine membranes to a yellow-brown for the modified membranes indicates that AgNPs were successfully immobilized on the membrane surface. The SEM-EDX spectra show that all the modified membranes had an apparent silver peak, while the pristine membranes did not, indicating the successful immobilization of AgNPs on PAA-modified membrane surfaces (Figure 6B). 

## 4. Conclusions

In summary, a substrate-independent method for immobilizing silver nanoparticles on polymer membrane surfaces by firstly modifying the membrane surface with functional groups (e.g., carboxylic groups), and then forming silver nanoparticles in situ by chemical reduction, was reported. Silver nanoparticles were successfully immobilized on polysulfone ultrafiltration membrane surfaces and were very stable. The modified membranes had very good anti-biofouling activity as demonstrated by disk diffusion and anti-biofouling tests, and the immobilized AgNPs were effective antibacterial agents against both Gram-negative and Gram-positive bacteria. The membrane anti-biofouling property can be regenerated as AgNPs on the membrane surface can be easily regenerated in situ. Furthermore, preliminary results of AgNPs immobilization on nylon, polypropylene microfiltration membranes, and reverse osmosis polyamide membranes suggest that this technique is able to be very widely employed for micro-, ultra-, nano-filtration, and RO membrane surfaces composed of various polymer membrane materials, such as polysulfone, polyether sulfone, polyvinylidene fluoride, nylon, polystyrene, polypropylene, and polyamide.

## Figures and Tables

**Table 1:** Experimental conditions for the membrane samples.

Sample ID	Pristine Membrane	2.5% AA Modified Membrane	AgNO_3_ Concentration (mM)	NaBH_4_ Concentration (mM)
**M0**	✓		-	-
**M0_AgNP**	✓		5	5
**PAA_PSf**		✓	-	-
**M1**		✓	1	5
**M2**		✓	3	5
**M3**		✓	5	5

**Table 2:** Atom Percentage of O and Ag on the Membrane Surfaces as Determined by XPS.

Sample ID	M0	PAA_PSf	M0_AgNP	M1	M2	M3
**O (At. %)**	8.4	12.2	-	-	-	-
**Ag (At. %)**	0	-	0.19	3.45	5.50	9.06

**Table 3:** The Content of Silver Loaded on the Membrane Surface Determined by ICP-OES and Estimated Time of Silver Release from the Membranes.

Sample ID	Total Ag Loaded (μg·cm^−2^)	Release Rate * (μg·cm^−2^·d^−1^)	Estimated Lasting Time (Days)	After Ag Regenerating (μg·cm^−2^)
**M1**	4.75	0.010	474	8.12
**M2**	10.88	0.020	543	13.92
**M3**	15.20	0.024	633	19.32

* The silver release rate is the average value between 7 and 14 weeks.

**Table 4:** Contact angles, membrane permeability and BSA rejection of the tested membranes.

Sample ID	M0	PAA_PSf	M1	M2	M3
**Contac t angle (°)**	62.0 ± 5.2	55.1 ± 1.6	54.3 ± 1.8	49.4 ± 6.3	50.4 ± 3.1
**Membrane permeability** **(L·m^−2^·h^−1^·bar^−1^)**	131.1 ± 2.4	63.2 ± 1.9	40.0 ± 3.6	36.2 ± 4.8	36.8 ± 1.3
**BSA rejection (%)**	75.8 ± 1.1	89.8 ± 0.9	92.3 ± 1.5	90.3 ± 0.6	91.7 ± 0.5

**Table 5:** Membrane Permeability for PP and Nylon Microfiltration, and Polyamide RO Membranes.

Sample ID	PP Membrane (Filtered at 0.2 bar)		Nylon Membrane (Filtered at 0.2 bar)		Polyamide RO Membrane (Filtered at 15 bar)	
	Pristine	With AgNPs	Pristine	With AgNPs	Pristine	With AgNPs
**Water permeability** **(L·m^−2^·h^−1^)**	616 ± 18	623 ± 23	484 ± 24	274 ± 16	39.3 ± 3.6	33.6 ± 4.5
